# Perceived stress and satisfaction with life following the 2023 earthquake in Türkiye: investigating the effects of irrational beliefs and hope

**DOI:** 10.3389/fpsyg.2025.1506669

**Published:** 2025-07-07

**Authors:** Esra Teke, Hüseyin B. Karaman

**Affiliations:** ^1^Malatya Turgut Özal University, Malatya, Türkiye; ^2^Guidance and Research Center, Bolu, Türkiye

**Keywords:** satisfaction with life, hope, irrational beliefs, perceived stress, earthquake

## Abstract

**Introduction:**

Recent earthquakes that struck Türkiye led to major destructions. Extensive body of research continues to investigate the psychological impacts of earthquake that can cause a broad range of public health problems. The present research aimed to examine perceived stress levels, irrational beliefs, hope levels and levels of satisfaction with life among individuals following the earthquake that occurred on 06 February 2023 in Türkiye.

**Methods:**

Four hundred and eighty two participants were enrolled in the research (Mage = 25.1 years, SD = 7.07). The research utilized Perceived Stress Scale, Dispositional Hope Scale, General Attitude and Belief Scale, and Satisfaction with Life Scale. For the data analysis, the SPSS 25 software package and the PROCESS macro version 4.2 were utilized.

**Results:**

The analyses showed significant correlations among the variables. The results for the model indicated that perceived stress levels were significantly associated with irrational beliefs, hope levels, and levels of satisfaction with life among individuals following the earthquake. In addition, perceived stress levels were indirectly associated with satisfaction with life through their relationships with irrational beliefs and hope.

**Discussion:**

The results highlight how perceived stress influences life satisfaction directly and indirectly through irrational beliefs and hope. These findings underscore the necessity for protective interventions to enhance psychological wellbeing after earthquakes.

## Introduction

Natural disasters including earthquakes are life events that occur around the world frequently. Such events usually strike suddenly and cannot be anticipated. They have the potential to be deathly by damaging environment and mental health with material and social losses (Iqbal and Sheikh, 2023; Patwary et al., 2023).

Two major earthquakes took place in Türkiye as well as Syria on 06 February 2023. Those earthquakes which led to major destructions represent the worst natural disaster in more than one century in terms of casualties (Kurt et al., 2023). Based on the official figures, 15 million people were affected by the earthquakes in Türkiye (World Health Organization, 2023). In the provinces struck by the earthquakes, 50,783 people passed away, and 115,353 people were injured (Disaster and Emergency Management Presidency, 2023). While emphasizing the physical damage, these figures show that earthquakes can also present a mental burden (Kobayashi et al., 2021) and lead to several problems (Çitak, 2023; Daneshvar et al., 2022; Djamaluddin and Umar, 2021; Kurt et al., 2023). Post-earthquake problems include anxiety, sleep disorders, dissociative disorders, and sexual dysfunction (Sönmez, 2022), PTSD (Alfuqaha et al., 2023), mental problems (Jafari et al., 2020). In addition, earthquakes can seriously disrupt mental health through economic and human losses (Ishaq et al., 2020; Kwon et al., 2023), cause individuals to become even more traumatized through their irrational beliefs (Djamaluddin and Umar, 2021), and lead to decreased satisfaction with life (Oishi et al., 2015). On the other hand, positive cognitive assessment of individuals following an earthquake may increase their quality of life (Kyutoku et al., 2012). Social support and hope levels can be effective in reducing the signs of stress (Çelik, 2023).

Available studies focus mainly on negative outcomes (Lu et al., 2023); however, it is critical to identify the protective and risk factors in the field of mental health to provide effective interventions (Chen et al., 2023). In this sense, it implies the need to investigate those factors in studies to be conducted following an earthquake. Indeed, the present research aimed to examine the mediation of hope and irrational beliefs in the relationship between perceived stress and satisfaction with life following the earthquake in question.

### Perceived stress and satisfaction with life

Stress is among normal reactions shown by individuals against challenging situations (Kaşikci et al., 2023). Individuals can exhibit diverse stress reactions following an earthquake (Kwon et al., 2023). Those reactions, seemingly normal at the beginning, can turn into serious mental health problems if measures are not taken (Daneshvar et al., 2022). In the literature, changing environmental conditions, psychological (Lu et al., 2023) and social support level, communication styles, and government policies (Gim and Shin, 2022), challenges during the reconstruction phase, level of preliminary knowledge and safety measures for earthquake (Rajabi et al., 2022), and emotions such as fear and guilt are among post-earthquake stress factors (Wang et al., 2020). Studies have shown that perceived stress can be a significant risk factor for negative mental health and an important determinant for low satisfaction with life (Ishaq et al., 2020).

Most of the available studies have found a correlation between perceived stress and low satisfaction with life (Extremera et al., 2009). Yet, many individuals preserve their high level of satisfaction with life despite increased stress levels and learn to adapt to recent changes successfully (Lee et al., 2016). Whereas, there are people who exhibit high levels of satisfaction with life despite their experience of intense stress following the earthquake, studies have failed to provide an adequate explanation of the matter. Hence, in-depth research is needed to explore complex interaction among several factors associated with the psychological and social outcomes of this earthquake which is described as one of the biggest earthquakes due to its destructiveness.

### Irrational beliefs and hope

According to the diathesis-stress model, individuals react differently to life events. Several events have been associated with increased psychological disturbances in individuals who are vulnerable to biological, situational, and psychological factors. In other words, stressful experiences interact with vulnerability may heighten the risk for several problems (Ingram and Luxton, 2005). Therefore, following an earthquake, stressors can interact with individual vulnerabilities, and are associated with poorer mental health outcomes and reduced wellbeing.

The cognitive vulnerability model suggests that irrational beliefs in individuals mediate the relationship between stressful life events and their emotional and behavioral reactions. According to the model, certain beliefs form vulnerability to those reactions (Beck, 2005). Within the model, explanations of the Rational Emotive Behavior Therapy (REBT) approach sheds light on the determination and understanding of the mediation mechanisms that influence such a relationship (Balkis and Duru, 2023).

REBT encourages individuals to think rationally and mitigate their irrational thoughts. REBT argues that psychological dysfunction is caused by individuals' beliefs about the events they have experienced. The REBT approach can be summarized within the ABC model which suggests that individuals' beliefs (B) about adversity (A) lead to emotional and behavioral reactions (C) (Ellis et al., 2010). REBT claims that irrational beliefs (B) assume a mediating role in the relationship between adverse life events (A) and emotional and behavioral consequences (Kaşikci et al., 2023). Here, the purpose is to enable different emotional or behavioral reactions by modifying the irrational beliefs.

In REBT, rational beliefs are flexible, not extreme, and consistent with reality. Irrational beliefs, on the other hand, are rigid, extreme, and inconsistent with reality (Sælid and Nordahl, 2017; Turner, 2016). Within this context, earthquake-related rational beliefs can be described as accepting the reality of the situation, perceiving that there are things which can be controlled and taking the necessary precautions (Turner, 2016). Irrational beliefs can be described as explaining the cause of earthquake with supernatural processes, accepting what has happened as fate and abandoning the practices of taking precautions (Bilik, 2019), and regarding earthquake as unpredictable and its damages as unavoidable (McClure et al., 2007). According to REBT, post-earthquake stressors can cause individuals to become even more traumatized through irrational beliefs as cognitive vulnerability factors (Djamaluddin and Umar, 2021). On the contrary, rational beliefs can become cognitive protective factors in comprehending the effects of post-earthquake stressors on individuals' satisfaction with life. Current studies have shown relationships between rational and irrational beliefs and mental health problems including depression and anxiety (Buschmann et al., 2018) as well as welfare indicators including psychological wellbeing (Kaşikci et al., 2023) and satisfaction with life (DiGiuseppe et al., 2018). Studies also provide evidence on the mediation of beliefs. For instance, Kaşikci et al. (2023) found the relationship between stress factors and ego resiliency to be mediated by rational and irrational beliefs. They also reported that irrational beliefs mediated between stress factors and psychological wellbeing. Based on the above-mentioned theoretical explanations and the previous findings, one would expect irrational beliefs to mediate between perceived stress and satisfaction with life.

It should be noted that despite being an important mediator between post-earthquake perceived stress and satisfaction with life, irrational beliefs are not the single factor. Another important concept in this relationship is hope. According to Snyder's hope theory (2002), hope is conceptualized as individual's ability to produce appropriate strategies to achieve the desired objectives and have the motivation to use those strategies. In stressful situations, acting in accordance with their objectives and finding new ways can determine individuals' reactions toward that stressful situation. Individuals can seek ways in line with their objectives and values in stressful situations and have the motivation to realize those ways. Thus, high hopes could be an important component in the mitigation of their stress signs by enabling them to cope with challenging circumstances and show resistance to those situations (Folkman, 2012; Çelik, 2023). Hope might therefore contribute to individuals' welfare indicators. Studies have found hope to be correlated with psychological wellbeing, gratitude, optimism (Kardas et al., 2019), psychological resilience (Gilman and Huebner, 2006) and satisfaction with life (Bailey et al., 2007; Kardas et al., 2019; Valle et al., 2006).

In addition, mitigating the irrational beliefs could help individuals develop the perception for being able to manage and control the stressors (Jones and Turner, 2022; Turner, 2016), which might increase their hope levels. For example, in their study which tested whether the knowledge and practical use of the ABC model as an important element of REBT increase the hope, Sælid and Nordahl (2017) found that REBT significantly mitigated the irrational thinking and significantly increased hope and that REBT had both instant and long-term effects. Based on these findings and considerations, we can assume that we can mitigate the irrational thinking to increase hope levels among individuals, therefore helping them cope with stressful life events. Thus, all these studies suggest that individuals with low levels of irrational thinking and high hope levels might possess improved stress coping skills, which could potentially be associated with levels of satisfaction with life. However, studies are needed to have an in-depth understanding of how hope and irrational beliefs are related to individuals' psychological status.

### The present study

Although post-earthquake damages and physical health outcomes have been extensively addressed in the literature, mental health outcomes could not be understood adequately (Ishaq et al., 2020). Yet, earthquakes have been associated with unprecedented public health issues including mental health problems (Kurt et al., 2023). As proven by the examples of earthquakes in Türkiye, victims might feel under stress following the earthquake (Iqbal and Sheikh, 2023). It does therefore present an obligation to address mental health outcomes of the earthquake (Daneshvar et al., 2022). Despite the availability of some studies on the subject matter, they focus rather on the negative outcomes of earthquake (Lu et al., 2023). Studies which address protective factors are needed to make effective interventions and mitigate the adverse effects of stress factors in the area of mental health. In this respect, REBT and hope offer valuable insights into the relationship between earthquakes and mental health. Exploring the irrational mindscapes and encouraging a change in them could help individuals cope with the post-earthquake stress, which might make individuals' adaptation easier, increase their welfare levels and improve their satisfaction with life. In addition to those beliefs, hope is an important factor which could contribute to this function. Indeed, hope encourages the individual to overcome challenging life events, mobilize their coping mechanisms to set themselves meaningful objectives. Within this context, hope can provide enlightening information on how individuals cope with post-earthquake stressful situations. Moreover, addressing the concept of hope could allow for identifying the protective factors that mitigate negative outcomes of stressful situations on mental health. Eventually, hope might make it possible to set appropriate objectives for individuals and the society within the context of earthquake, develop strategies in line with those objectives, and take functional steps in using those strategies.

In summary, although earthquakes are regarded as an important public health issue including the mental health problems, further research are required to have a sufficient understanding of the matter. REBT and hope could provide a framework for reducing the negative psychological effects of earthquake. Future studies can allow for noticing the encouraging mechanisms and protective factors in the mitigation of earthquake's negative impact. Moreover, perceived stress levels of individuals victimized by earthquake could be assessed in several areas to minimize the effects of this experience, and individuals can be encouraged to act in line with their objectives and purposes. Findings to be obtained can be used to develop effective interventions and to improve welfare levels of individuals and the society.

**Figure 1:**
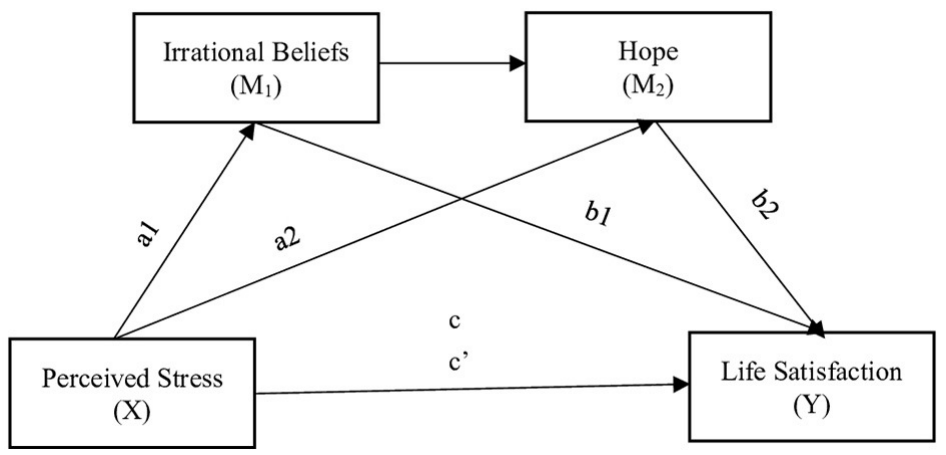
Model constructed with the variables; perceived stress, satisfaction with life, irrational beliefs, and hope. *a1*, Effect of perceived stress on irrational beliefs; *a2*, Effect of perceived stress on hope; *b1*, Effect of irrational beliefs on life satisfaction; *b2*, Effect of hope on life satisfaction; *c*, Total effect of perceived stress on life satisfaction; *c'*, Direct effect of perceived stress on life satisfaction, controlling for mediators.

In the present study, levels of satisfaction with life among individuals who directly or indirectly (through social media, television, internet, news reports) experience the earthquake that took place on 06 February 2023 were examined with respect to their perceived stress, irrational beliefs, and hope levels. A structural equation model was established with these variables. In the model, the predictive variable included perceived stress levels, while satisfaction with life was the predicted variable, and irrational beliefs and hope served as the mediator variables. The research model is provided in Figure 1.

The following hypotheses were developed in the study:

H1: Perceived stress is significantly associated with irrational beliefs, hope, and life satisfaction.
H2: Perceived stress is indirectly associated with life satisfaction through irrational beliefs and hope.

## Method

### Participants

Sample size adequacy is crucial for determining any potential effects among variables. For a mediation analysis, a sample size of 115 to 285 participants is enough to identify the effect among the variables (Fritz and MacKinnon, 2007). Data were collected from as many participants as possible in this analysis (*N* = 482). This number is way beyond the specified range, which reduces chances of inconclusive results.

The long-term negative consequences of an earthquake on individuals can affect their psychological wellbeing, leading to traumatic effects that can persist for years (Lu et al., 2023). In addition to causing serious psychological effects on people living in the region, traumatic experiences such as earthquakes can also have negative mental consequences for individuals who are exposed to the earthquake as a secondary event and are at risk of earthquakes (Kwon et al., 2023). Earthquakes that have occurred in Türkiye since February 6, 2023, are seen as a social trauma that may be associated with the entire society, not just individuals who directly experience the earthquakes and suffer various losses (Biçakci and Okumuş, 2023). In the studies carried out, it is logical for the research sample to consist of people living in both areas affected and not affected by the earthquake (Çitak, 2023). This aspect was taken into consideration in the study.

As presented in Table 1, the study enrolled 391 women (M_age_ = 25.1 years, SD = 7.07, range = 18 to 49) and 91 men (M_age_= 26.9 years, SD = 8.34, range = 18 to 44) who were selected with convenience sampling method and located in Türkiye including the 11 provinces affected by the earthquake. The most frequently reported demographic categories were bachelor's degree (*n* = 313) and those who were in other provinces at the time of the earthquake (*n* = 203).

**Table 1:** Participant characteristics (*n* = 482).

**Variable**	**Level**	**Frequency**	**Percent**
Gender	Male	91	18.9
	Female	391	81.9
Educational level	High school or below	22	4.6
	Associate's degree	77	16.0
	Bachelor's degree	313	64.9
	Master's degree	41	8.5
	Doctorate	29	6.0
Provinces where the participants were located at the time of earthquake	Those who were in one of the 11 affected provinces at the time of earthquake	203	42.1
	Those who were in other provinces at the time of earthquake	279	57.9

During our investigation, the data collection process was conducted under extraordinary circumstances. In areas still experiencing the repercussions of the earthquake, traditional face-to-face data collection methods were unfeasible. This was primarily due to the persistence of online education at numerous universities and the displacement of individuals to safer regions. Consequently, we utilized social media platforms, including WhatsApp, Instagram, and Facebook, to collect the required data. Research data were gathered through a web-based survey that included study measures and sociodemographic questions. The survey participants were submitted an informed consent form before completing the survey. Participation was on voluntary basis, and the survey was completed anonymously. It took about 15 min to complete the survey. Due to the survey design in which participants had to answer all questions, there were no participants who rejected participation or did not complete the survey. Data collection was performed between 18 and 31 December 2023. The research has received approval from the ethical committee of a university in Türkiye and conducted in accordance with the 1964 Helsinki Declaration.

### Materials

#### Perceived stress scale (PSS)

The scale used to measure the perceived stress levels of the individuals in the study following the earthquake was developed by Cohen et al. (1983). The scale comprises of 14 items and two subscales. Seven items measure the perceived lack of self-efficacy (e.g. “In the last month, how often have you felt that you were unable to control the important things in your life?”) whereas seven items measure the perceived helplessness (e.g. “In the last month, how often have you been angered because of things that were outside of your control?”). All items are rated on a 5-point Likert scale as “Never (0)” to “Very Often (4).” Seven of the items are reverse-coded. Scale scores vary between 0 and 56. Higher scores on the scale indicate higher levels of perceived stress. Eskin et al. (2013) assessed the validity and reliability of PSS for the Turkish sample and found an internal consistency coefficient of 0.84 as well as a test-retest reliability coefficient of 0.87. The study demonstrated that the scale has strong internal consistency estimates and good psychometric properties for the Turkish sample. In the present study, we found that the internal consistency coefficient of the scale was 0.83.

#### General attitude and belief scale

Used to measure individuals' irrational beliefs, the scale was developed by Lindner et al. (1999). The scale is composed of 26 items (e.g., “It's awful to have hassles in one's life and it is a catastrophe to be hassled”). All items are rated on a 5-point Likert scale from strongly agree (5) to strongly disagree (1). Higher scores on the scale indicate a higher level of irrational belief. Artiran (2019) found an internal consistency of 0.84 for the scale and showed the scale to be valid and have strong internal consistency estimates for the Turkish sample. The scale in this study showed an internal consistency coefficient of 0.94.

#### Satisfaction with life scale

Satisfaction with life scale was utilized to identify individuals' perceptions of satisfaction with their lives (Diener et al., 1985). The 5-item scale with one factor is rated on a 5-point Likert scale from strongly disagree (1) to strongly agree (5) (e.g., “I am satisfied with my life”). On the scale, higher scores indicate a greater level of satisfaction with life. Dağlı and Baysal (2016) reported a Cronbach's alpha internal consistency coefficient of 0.88 and a test-retest reliability coefficient of 0.97 for the scale. They also showed the scale to provide accurate model statistics for the Turkish sample. In this study, internal consistency coefficient of the scale was found to be 0.85.

#### Dispositional hope scale

Developed by Snyder et al. (1991), the scale was used to measure hope levels of the individuals. The scale consists of 12 items and two subscales. Each of the subscales which are alternative pathways thinking (e.g., “Even when others despair, I think I can find a way to solve the problem”) and agency thinking (e.g., “I achieve the goals that I set for myself”) is measured with four items. One of these four items is about the past, two about the present, and one about the future. Other four items are distractors which are not related to hope. The scale is rated from Definitely false (1) to Definitely true (8). The lowest score on the scale is 8, while the highest is 64. Higher scores mean higher hope levels. Tarhan and Bacanli (2015) found an internal consistency coefficient of 0.84 and a test-retest reliability coefficient of 0.86 for the scale. Their analysis concluded that the scale could be a useful tool for the Turkish context. In this study, the scale showed an internal consistency coefficient of 0.87.

### Data analysis

Several data analyses were performed to examine how irrational beliefs and hope mediate the relationship between perceived stress and satisfaction with life. Initially, preliminary analyses were conducted including the normality assumption, observed scale characteristics, and correlation analysis. The normality assumption was assessed using skewness and kurtosis scores, and their cutoff values. For the normality assumption, kurtosis and skewness scores, and their cutoff values were investigated with scores below |1|. The values were found to be acceptable for normal distribution (Mayers, 2013). Next, Pearson's correlation analysis was performed to examine the correlations among the study variables. Following the evaluation of the preliminary analyses, serial mediation analysis was used to analyze the mediation model showing the role of irrational beliefs and hope in the relationship between perceived stress and satisfaction with life. For this intermediary analysis, Hayes' Model-6 was employed using the Process Macro. Additionally, the bootstrap method was applied with a sample of 5,000 iterations to compute 90% confidence intervals (CI) for indirect effects (Hayes, 2018). In interpreting the results of the model established in the analysis, standardized effects were categorized as low for values approximating 0.01, moderate for values near 0.09, and high for values around 0.25 (Preacher and Kelley, 2011). The analysis utilized SPSS version 25 and the Process Macro.

## Findings

### Preliminary analyses

Based on the results for the model constructed for the present research, preliminary analyses were conducted for normality values of the data, intervariable correlations, and mean scores from the scales. Results of the preliminary analyses are provided in Table 2.

**Table 2:** Observed scale characteristics and correlation results.

**Scale**	**1**	**2**	**3**	**4**
1. Perceived stress	–	−0.34^**^	0.51^**^	−0.42^**^
2. Hope		–	−0.55^**^	0.43^**^
3. Irrational beliefs			–	−0.56^**^
4. Satisfaction with life				–
Range	20–69	21–64	27–93	5–25
Mean	44.88	48.43	67.92	15.76
Standard deviation	8.11	9.21	19.17	4.32
Skewness	0.02	−0.56	0.10	−0.17
Kurtosis	0.09	−0.02	−0.41	−0.22
Internal reliability	0.83	0.87	0.94	0.85

** *p* < 0.001.

According to Table 2, mean scores from the scales had skewness and kurtosis values between −0.56 and 0.41, and the distribution was normal (Mayers, 2013). As for the reliability of the scales, their Cronbach's alphas ranged between 0.83 and 0.94. Based on the correlation results, there was a moderately significant negative correlation between perceived stress and hope (*r* = −0.34, *p* < 0.001), a highly significant positive correlation between perceived stress and irrational beliefs (*r* = 0.51, *p* < 0.001), and a moderately significant negative correlation between perceived stress and satisfaction with life (*r* = −0.42, *p* < 0.001). There was a highly significant negative correlation between hope and irrational beliefs (*r* = −0.55, *p* < 0.001) and a highly significant positive correlation between hope and satisfaction with life (*r* = 0.43, *p* < 0.001). A highly significant negative correlation was found between irrational beliefs and satisfaction with life (*r* = −0.56, *p* < 0.001).

**Figure 2:**
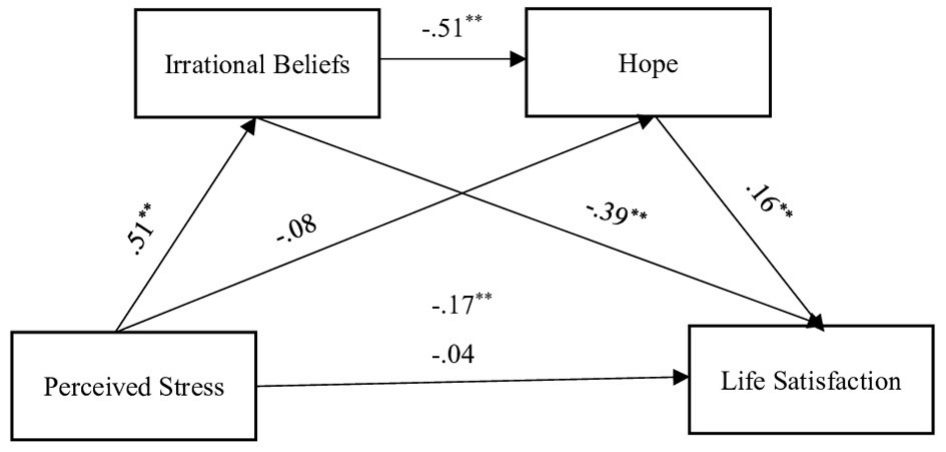
Proposed model indicating the standardized associations between variables. **p < 0.001.

Following the preliminary analyses, further analyses were performed in line with the model in Figure 1. Indirect effect of perceived stress on satisfaction with life through irrational beliefs and hope was also examined in the research. Results for the model are shown in Figure 2. Results for the direct and indirect effects in the model are provided in Tables 3, 4, respectively.

**Table 3:** Unstandardized total, direct, and indirect effect for the life satisfaction model.

**Path**	**Effect**	**SE**	**BootLLCI**	**BootULCI**
Total effect	−0.22	0.02	−0.26	−0.19
Direct effect	−0.09	0.02	−0.12	−0.05
Total indirect effect	−0.13	0.02	−0.16	−0.10
PSS → IRB → LS	−0.11	0.01	−0.13	−0.08
PSS → Hope → LS	−0.01	0.00	−0.01	−0.00^*^
PSS → IRB → Hope → LS	−0.02	0.01	−0.06	−0.01

PSS, Perceived Stress Scale; IRB, Irrational Beliefs; LS, Life Satisfaction; SE, Standard Error; BootLLCI, Lower-Level Confidence Interval, BootULCI; Upper-Level Confidence Interval. The number of bootstrap samples for percentile bootstrap confidence intervals: 5,000.

* The BootULCI value is presented as −0.00 due to rounding; however, the precise value is −0.0009.

**Table 4:** Completely standardized indirect effect size(s) for the substance life satisfaction model.

**Path**	**Effect**	**SE**	**BootLLCI**	**BootULCI**
Total effect	−0.25	0.03	−0.30	−0.20
PSS → IRB → LS	−0.20	0.03	−0.24	−0.15
PSS → Hope → LS	−0.01	0.01	−0.03	−0.00^*^
PSS → IRB → Hope → LS	−0.04	0.01	−0.06	−0.02

SE, Standard Error; BootLLCI, Lower-Level Confidence Interval; BootULCI, Upper-Level Confidence Interval; Number of bootstrap samples for percentile bootstrap confidence intervals: 5,000.

* The BootULCI value is presented as −0.00 due to rounding; however, the precise value is −0.0018.

### Findings on hypothesis 1

An analysis of the standardized effects illustrated in Figure 2 revealed that perceived stress was significantly associated with irrational beliefs (β = 0.51, *p* < 0.05) and life satisfaction (β = −0.17, *p* < 0.05). The findings further indicated that irrational beliefs were significantly associated with both hope (β = −0.51, *p* < 0.05) and life satisfaction (β = −0.39, *p* < 0.05). Moreover, hope was found to be significantly associated with life satisfaction (β = 0.16, *p* < 0.05). These results provide support for Hypothesis 1.

### Findings on hypothesis 2

Examining the non-standardized total, direct and indirect effects in the life satisfaction model as presented in Table 3, it was determined that perceived stress predicted life satisfaction through irrational beliefs (β = −0.11, *p* < 0.05). Similarly, perceived stress was found to significantly predict life satisfaction through hope (β = −0.01, *p* < 0.05). Furthermore, perceived stress was observed to significantly predict life satisfaction through both irrational beliefs and hope (β = −0.02, *p* < 0.05).

An analysis of Table 4, Figure 2 revealed that the standardized values of indirect effects demonstrated that perceived stress was indirectly associated with life satisfaction through irrational beliefs (*K2* = −0.20). Similarly, perceived stress showed a significant indirect association with life satisfaction via hope (*K2* = −0.01). Additionally, perceived stress significantly predicted life satisfaction through both irrational beliefs and hope (*K2*= −0.04).

## Discussion

The recent earthquake in Türkiye led to major destructions and an increasing number of casualties (Iqbal and Sheikh, 2023). Exhaustive body of research continues to investigate the psychological effects on mental health and wellbeing caused by this earthquake which could lead to unprecedented public health issues including mental health problems (Kurt et al., 2023). The main focus of the current research was to examine the associations between the perceived stress following an earthquake and individuals' the irrational beliefs, hope levels, and satisfaction with life. Purpose of this research was to investigate the direct effect of perceived stress levels following the earthquake on irrational beliefs, hope, and satisfaction with life and to explore the indirect effect of perceived stress on life satisfaction through irrational beliefs and hope. Accordingly, it was aimed to study the inter-variable correlations to gain a deep understanding of the psychological effects of the earthquake and to identify encouraging mechanisms and protective factors for reducing the negative effects associated with the earthquake. The findings indicated that post-earthquake perceived stress levels were negatively correlated with hope and satisfaction with life and positively correlated with irrational beliefs. These findings are consistent with the literature demonstrating the detrimental effects of post-earthquake stress on mental health outcomes (Daneshvar et al., 2022; Ishaq et al., 2020; Kwon et al., 2023). Earthquakes have been described as a serious stress factor due to economic and human losses and their associations with negative outcomes on mental health and wellbeing levels have been shown by increasing body of research (Alfuqaha et al., 2023; Çitak, 2023; Daneshvar et al., 2022; Jafari et al., 2020; Kobayashi et al., 2021; Kurt et al., 2023; Sönmez, 2022). Changing environmental conditions, challenges during the reconstruction process, human losses, and emotions including fear and guilt are among important post-earthquake stress factors (Lu et al., 2023; Wang et al., 2020). Experiences through these stress factors can affect individuals' rational assessments, therefore suppressing their rational beliefs with respect to taking the required measures against earthquakes, realizing their current resources, and taking the necessary action (Bilik, 2019; McClure et al., 2007). For instance, previous studies have underlined the relationship between high stress levels and irrational beliefs such as catastrophizing and overgeneralization (Buschmann et al., 2018) and demonstrated a relationship between perceived stress and irrational beliefs (Kaşikci et al., 2023). Studies highlight perceived stress as a risk factor for adverse mental health (Ishaq et al., 2020) and its possible effect on reduced satisfaction with life (Extremera et al., 2009; Ishaq et al., 2020; Oishi et al., 2015). Experiences can cause rational beliefs to be suppressed and irrational beliefs to prevail and be associated with negative perceptions of environmental inhabitability, individual's life skills, benefit of life, and individual's assessment about appreciating the life, which are considered among criteria of satisfaction with life. In addition to damaging the environment and mental health, earthquake's potential deathliness might increase perceived stress levels of individuals and reduce satisfaction with life among earthquake victims and cause people to develop signs of hopelessness (Iqbal and Sheikh, 2023). These outcomes could lead to adverse mental health outcomes (Kwon et al., 2023). Yet, hope can be a protective factor that mitigates negative effects of stressful situations on mental health (Çelik, 2023). Indeed, individuals who seek and use new ways for their objectives could minimize the effects of the stressful events they have experienced (Folkman, 2012; Çelik, 2023). The findings from the literature contribute to our understanding of the psychological consequences associated with earthquakes. They also emphasize the need for interventions to reduce the negative effects of post-earthquake stress and underscore the importance of promoting mechanisms and protective factors.

The findings of this study reveal the indirect effect of perceived stress on life satisfaction mediated by irrational beliefs and hope, thereby offering significant insights into post-earthquake mental health processes. The findings emphasize that perceived stress decreases life satisfaction by fostering an increase in irrational beliefs. For instance, Kaşikci et al. (2023) found stress factors to be indirectly associated with both ego resiliency and psychological wellbeing through irrational beliefs. Kyutoku et al. (2012) found a negative relationship between negative cognitive assessments and quality of life. Hamarta et al. (2009) showed in their study with undergraduates that irrational beliefs of individuals were negatively associated with their coping behaviors in stressful situations. These findings underscore the significant influence of cognitive distortions, which individuals develop in response to perceived stress, on mental health. Furthermore, our study identified a negative association between perceived stress on hope and elucidated the essential function of hope in enhancing life satisfaction. This result highlights the important role of hope between perceived stress and satisfaction with life among individuals following the earthquake and suggests that it has a notable effect in the context of earthquake. Findings also suggest that individuals with high levels of hope are more likely to achieve more positive psychological outcomes despite intense stress following the earthquake. In this sense, the findings are also consistent with the studies demonstrating the protective effects of hope in the relationship between perceived stress and better psychological outcomes. Nouzari et al. (2019) found that hope in addition to post-traumatic growth had a positive effect on individuals' psychological conditions following the experience of stressful situations. Bailey et al. (2007) found hope to be a good predictor of satisfaction with life. Similarly, studies have shown hope to be correlated with psychological wellbeing, gratitude, optimism, psychological flexibility, meaning of life, post-traumatic growth, and satisfaction with life (Gilman and Huebner, 2006; Kardas et al., 2019; Valle et al., 2006). Taken together, our study revealed the role of irrational beliefs and hope as serial mediators in the relationship between perceived stress and life satisfaction. The findings suggest that perceived stress is initially associated with higher levels of irrational beliefs, which are, in turn, linked to reduced hope. This decrease in hope is subsequently associated with lower life satisfaction. The serial mediation model offers a comprehensive framework for understanding how cognitive distortions, developed in response to stress, are associated with reduced levels of hope and, in turn, with lower life satisfaction. This mechanism is also consistent with the principles REBT. The REBT encourages individuals to engage in rational thinking and diminish irrational thought patterns, arguing that such rational cognition can positively influence mental health outcomes (Balkis and Duru, 2023; Ellis et al., 2010). In their study, Sælid and Nordahl (2017) found that REBT significantly reduced irrational thinking and enhanced hope, with both immediate and long-term effects. Similarly, Rezaeisharif et al. (2021) that cognitive restructuring interventions effectively reduce irrational beliefs and hopelessness. Research indicates that an increase in irrational beliefs may be associated with negative health outcomes (Buschmann et al., 2018; Noormohamadi et al., 2022). Furthermore, the reduction of such beliefs has been shown to positively influence mental health (Kyutoku et al., 2012). Empirical evidence supports the association between beliefs and life satisfaction (DiGiuseppe et al., 2018) and suggests that a decrease in irrational beliefs significantly enhances hope (Rezaeisharif et al., 2021; Sælid and Nordahl, 2017).

When considered in conjunction with REPT, it becomes evident that irrational beliefs significantly influence mental health and stress management. This perspective is crucial for understanding how individuals' levels of hope can be sustained through cognitive restructuring processes. The findings of this study reveal how irrational beliefs and hope function as a serial mediation mechanism in the relationship between perceived stress and life satisfaction. Notably, the high level of stress experienced following an earthquake may increase individuals' irrational beliefs and leading them to interpret events in a more catastrophic and hopeless framework (Turner, 2016). This irrational mode of thinking, on the other hand, reduces individuals' levels of hope, undermines their positive expectations for the future and ultimately decreases their life satisfaction (DiGiuseppe et al., 2018). These findings, which align with the principles of the REBT, suggest that cognitive-behavioral interventions aimed at addressing irrational beliefs may effectively enhance individuals' life satisfaction by increasing their hope levels. Thus, implementing post-earthquake initiatives that focus on the mental health of survivors, addressing irrational beliefs, and foster hope can be an effective approach to enhancing overall wellbeing.

It is essential to consider that the psychological processes examined in our study may have differential effects on individuals based on gender. The literature suggests that the impact of stress on irrational beliefs and hope may vary between genders, with women potentially being more susceptible to stressors (Torpus et al., 2024). In this context, the gender distribution in our study is a critical factor to consider when interpreting the findings.

In our study, it was observed that the proportion of female participants exceeded that of male participants. The literature indicates that women are more inclined than men to engage in self-report scales (Curtin et al., 2000; Singer et al., 2000). The nature of online surveys, which are predicated on information sharing, may contribute to higher participation rates among women, whereas men may engage in such studies at lower frequencies (Smith, 2008). In addition, research demonstrates that women exhibit greater sensitivity to stressors and report higher levels of psychological distress compared to men in extraordinary circumstances, such as earthquakes (Karanci et al., 1999; Sumer et al., 2005). It has been determined that women's emotional and cognitive evaluation processes differ, and their subjective and physiological responses to stressful events are more pronounced than those of men (Nolen-Hoeksema, 2001; Schienle et al., 2005).

The observed differences may also be reflected in the findings of our study. The tendency of female participants to report higher levels of perceived stress may have contributed to the stronger mediating role of irrational beliefs on stress and life satisfaction. The differentiation in the effects of stress on cognitive assessment processes between genders may also influence the emergence of irrational beliefs. It is established that men are more inclined toward problem-oriented strategies in coping with stress, whereas women tend to engage in more emotional evaluation processes (Erözkan, 2004). Consequently, the association between irrational beliefs and stress may be more pronounced among female participants.

Similarly, the mediating effect of hope between stress and life satisfaction may differ according to gender differences. The literature indicates that women are more inclined to utilize social support mechanisms and employ hope more frequently as a coping strategy (Yilmaz and Sahin, 2020). The tendency of women to use hope as a resource in managing stress may have contributed to the more pronounced mediating role of hope observed in the findings of our study.

The findings indicate that gender may serve as a significant variable in post-earthquake psychological processes. Variations in coping mechanisms between men and women can elucidate the differential effect of stress on irrational beliefs and hope. This perspective is crucial for comprehending the influence of gender-based psychological processes on post-disaster mental health. Future research should focus on examining the effect of gender on psychological variables in greater detail. Furthermore, designing post-disaster mental health services that consider individual differences in coping mechanisms may enhance the development of more effective interventions aimed at improving psychological wellbeing.

### Conclusion and limitations

The present study demonstrated that post-earthquake perceived stress was negatively associated with individuals' levels of satisfaction with life. The study further substantiated the REBT approach by demonstrating that irrational beliefs and hope serve as mediators between perceived stress and life satisfaction. With outputs showing that hope is a potential force acting as a protective factor in the relationship between perceived stress and satisfaction with life, the study also contributes to the cognitive-motivational construct related to hope. By this means, it highlights the important role of irrational beliefs in shaping the mental health in stressful situations and calls attention to hope as a supporting factor in mental health. To sum up, the present study provides evidence for the purpose of increasing the wellbeing levels, encourages the appropriate interventions while contributing to our understanding of earthquake-related psychological mechanisms.

Whereas, this study offered an important argument for research and practice, it should be assessed considering a few limitations. Firstly, the cross-sectional design of the research precludes establishing causations. Longitudinal studies are needed to test time-dependent changes of the model tested. The data are limited to self-reports of the participants. Future studies could benefit from multiple techniques to investigate the study variables. Qualitative and mixed research methods can also be utilized to achieve more extensive results. In addition, the data collection process was conducted under extraordinary circumstances. During the period when the effects of the earthquake persisted, face-to-face data collection methods were not feasible, and thus, the data were collected via social media platforms (eg., WhatsApp, Instagram, Facebook). However, this approach may have restricted the participation of individuals without access to or who do not actively use social media. Future studies are advised to incorporate alternative techniques such as telephone interviews or face-to-face surveys, in addition to online data collection methods. Besides, it was observed that the proportion of female participants exceeded that of male participants in our study. The literature indicates that women are more inclined to engage in self-report scales than men (Curtin et al., 2000; Singer et al., 2000). This discrepancy may have resulted in an imbalance in gender representation within the study, thereby limiting the generalizability of the findings. Future research should develop sampling strategies to ensure a more equitable representation of both female and male participants, thereby enhancing data balance. This study enrolled participants aged between 18 and 49 years. Future studies could use diverse samples to examine the relationships among the variables. The present research was conducted with participants who were and were not in the disaster zone at the time of the earthquake. Studies could further assess victims and non-victims separately and compare the results. In addition, pre- and post-earthquake factors that affect mental health of the participants could be examined individually.

## Data Availability

The datasets generated during and/or analyzed during the current study are available from the corresponding author on reasonable request.
